# Structural and biochemical rationale for Beta variant protein booster vaccine broad cross-neutralization of SARS-CoV-2

**DOI:** 10.1038/s41598-024-52499-1

**Published:** 2024-01-23

**Authors:** Eduardo M. Bruch, Shaolong Zhu, Lisa Szymkowicz, Taylor Blake, Tara Kiss, D. Andrew James, Alexey Rak, Kartik Narayan, Matthew T. Balmer, Roman M. Chicz

**Affiliations:** 1 Sanofi, Vitry-sur-Seine, France; 2 Sanofi, Toronto, ON Canada; 3 Sanofi, Swiftwater, PA USA; 4 Sanofi, Cambridge, MA USA; 5 Sanofi, Waltham, MA USA

**Keywords:** Biochemistry, Structural biology

## Abstract

Severe acute respiratory syndrome coronavirus 2 (SARS-CoV-2), responsible for the COVID-19 pandemic, uses a surface expressed trimeric spike glycoprotein for cell entry. This trimer is the primary target for neutralizing antibodies making it a key candidate for vaccine development. During the global pandemic circulating variants of concern (VOC) caused several waves of infection, severe disease, and death. The reduced efficacy of the ancestral trimer-based vaccines against emerging VOC led to the need for booster vaccines. Here we present a detailed characterization of the Sanofi Beta trimer, utilizing cryo-EM for structural elucidation. We investigate the conformational dynamics and stabilizing features using orthogonal SPR, SEC, nanoDSF, and HDX-MS techniques to better understand how this antigen elicits superior broad neutralizing antibodies as a variant booster vaccine. This structural analysis confirms the Beta trimer preference for canonical quaternary structure with two RBD in the up position and the reversible equilibrium between the canonical spike and open trimer conformations. Moreover, this report provides a better understanding of structural differences between spike antigens contributing to differential vaccine efficacy.

## Introduction

The COVID-19 pandemic caused by the SARS-CoV-2 virus gave rise to unprecedented efforts to rapidly study the virus and develop vaccines to protect against severe disease, hospitalization, and death. Since the declaration of the global health crisis, over fifty vaccines have been available under Emergency Use Authorization (EUA) or standard Marketing Authorization by regulatory agencies^1^. The first administered vaccines were based on the ancestral (Wuhan Hu-1) spike antigen using mRNA, Adenovirus, inactivated virus, and recombinant protein platforms. As the pandemic progressed, variants of concern (VOC) adapted to escape immune detection and emerged around the world differing structurally by amino acid substitutions throughout the viral genome. Several outbreaks occurred, each being defined by a sequence lineage (Alpha, Beta, Gamma, Delta, Omicron). As each lineage circulated, it became apparent that primary immunization with the ancestral spike elicited neutralizing antibodies of limited durability and booster vaccines were adopted to provide cross-neutralization against the emerging VOC and to extend the durability of the primary immunization.

Certain phylogenetic mutations have carried over across the VOC including three RBD amino acid substitutions, K417N, E484K, and N501Y providing new epitopes compared to the ancestral spike antigen. The SARS-CoV-2 evolution continued adapting to the human host extending to the current Omicron lineage which in general is less pathogenic yet more transmissible compared to the ancestral strain. Primary immunization Phase 3 efficacy studies with the ancestral spike antigen demonstrated good protection against the first VOC. However, this protection dropped significantly in late 2021 with the emergence of the Omicron variants^2^. First generation booster vaccines initially provided encouraging protection, but the circulating neutralizing antibodies appeared to wane at 4 to 5 months^3,4^, leading regulatory agencies to employ a strain chasing strategy for the development of future vaccines contributing to the nearly 200 vaccine candidates in clinical development^1^.

Sanofi initiated development of a B.1.351 (Beta) variant strain vaccine at the beginning of 2021. The Beta containing bivalent vaccine candidate is the only primary vaccine to demonstrate efficacy against all circulating VOC including Omicron (BA.1, BA.2, BA.4/5 and BF.7) in a Phase 3 double blind placebo-controlled efficacy trial^5^. Three formulations (monovalent ancestral and Beta spike and a bivalent combination of the two) were developed in parallel for both primary^5–9^ and booster vaccine indications^10–13^. As the pandemic continued, the general population became progressively more exposed to circulating VOC and booster vaccine development became the priority. The monovalent Beta booster vaccine has demonstrated superior cross-neutralizing antibody titres and durability for up to one year in both preclinical NHP studies^10,11^ and Phase 2/3 clinical trials comparing first generation ancestral strain mRNA and recombinant protein booster vaccines^12,13^.

Here we describe protein characterization of the prefusion Beta spike antigen engineered with a trimerization domain used in the approved Sanofi booster vaccine to support the superior immunogenicity observed in preclinical and clinical studies^14^. Biochemical and structural analyses (including ligand binding assays) were performed to characterize the reversible temperature dependent conformational transitions between open trimer and multiple canonical structures observed for trimerized protomers of the SARS-CoV-2 spike antigen. These studies allowed better understanding of the effect of low temperature storage on vaccine stability and immunogenicity.

## Results

### Canonical vs open-trimeric spike

**Figure 1:**
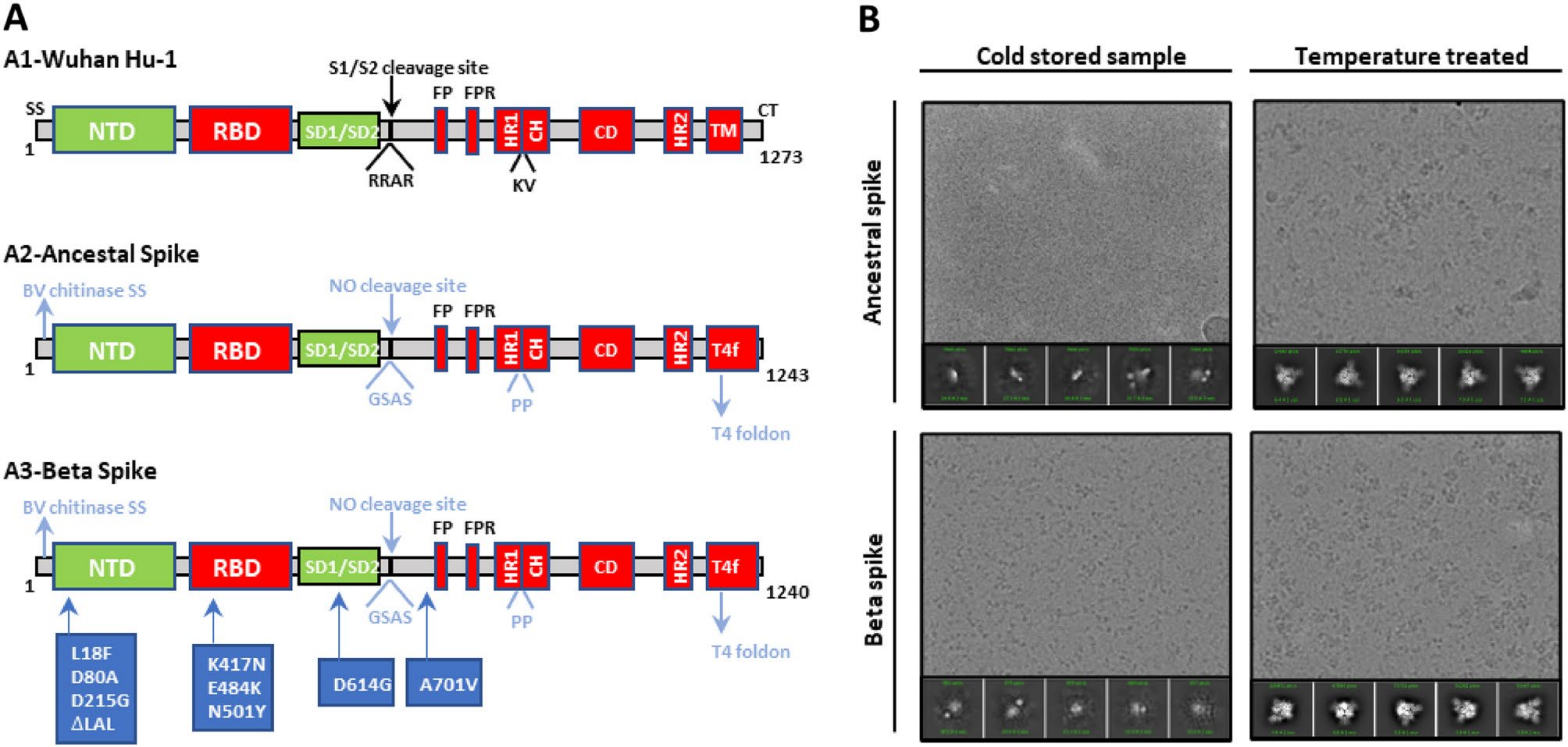
Ancestral and Beta spike antigens: Constructs and cryo-EM particle visualization. (**A**) Schematic of SARS CoV-2 S-glycoproteins. Structure of native spike (A1) from the Wuhan Hu-1 isolate, ancestral spike, with amino acids and region modified in cyan (A2), Beta spike antigen with additional modifications in blue boxes (A3). The numbering of the deletions and point mutations is based on the Wuhan Hu-1 full-length protein sequence. SS, signal sequence; NTD, amino terminal domain (14–305); RBD, receptor binding domain (331–527); SD1/SD2, C-terminal domains 1 and 2 (528–685); S1/S2 protease cleavage site (685); FP, fusion peptide (816–855); FPR, fusion peptide region (856–911); HR1, heptad repeat 1 (912–984); CH, central helix (985–1034); CD, connector domain (1076–1141); HR2, heptad repeat 2; TM, transmembrane domain; CT, cytoplasmic tail. (**B**) Cryo-EM representative images and most populated 2D-classes for both antigens after long storage at 4ºC and after temperature treatment (37ºC for 1 day).

The first Sanofi SARS-CoV-2 recombinant vaccine candidate was based on the spike glycoprotein gene sequence from the ancestral Wuhan YP_009724390.1 strain (Wuhan Hu-1) while the second one was based on the B.1.351 variant (GISAID Accession EPI_ISL_1048524). They are referred to as ancestral and Beta spike antigen, respectively. The genes were modified to optimize expression and secretion using the proprietary Sanofi Baculovirus expression system. The first 39 residues were replaced by a 54 bp sequence coding for the baculovirus chitinase signal peptide. To avoid cleavage within the S1-S2 subunits, the furin protease cleavage site residues RRAR were replaced by GSAS. To stabilize both proteins in the prefusion conformation two proline substitutions of residues 986 and 987 were performed^15,16^. The region encoding for the transmembrane helix and cytosolic tail were replaced by a 27 amino acid C-terminal T4 bacteriophage fibritin foldon domain to ensure protein trimerization. (Fig. 1A and Supplementary Fig. 1).

Recombinant spike antigen was previously reported to adopt an open trimeric conformation when stored for prolonged periods of time at 4 °C. This open trimeric conformation can interconvert reversibly to a canonical trimeric conformation when incubated at 25 °C or 37 °C^17,18^. To characterize the structural properties of the recombinant antigens, cryogenic electron microscopy (cryo-EM) analyses were performed on samples stored at 4 °C and after incubation at 37 °C. For cold stored samples, no evidence of canonical spike particles was observed either in the micrographs or in the 2D-classes (Fig. 1B). For both antigens, the canonical spike trimeric conformation was observed in the micrographs and the 2D-classes after incubation at 37 °C.

Size exclusion chromatography analyses of ancestral and Beta spike antigens showed similar elution profiles consistent with highly purified proteins migrating as single peaks (Extended Data Fig. 1A). The observed elution time corresponded to trimeric forms with no evidence of monomeric species or degradation fragments detected, consistent with the > 90% purity release specifications. The SEC chromatograms show later elution times for antigens incubated at 37 °C when compared to cold stored antigens (32.54 min. vs 32.07 min. for Beta and 31.51 min vs 30.70 min for ancestral spike antigen). A detailed analysis of the corresponding profiles showed narrower and more symmetric peaks for antigens incubated at 37 °C compared to broader and more asymmetric peaks for cold storage samples. Both the elution times and peak shapes were consistent with a more compact antigen conformation after incubation at 37 °C. (Extended Data Fig. 1A).

For both ancestral and Beta trimer, nanoDSF results (Extended Data Fig. 1B and C) showed a first folding transition at lower temperatures for cold stored when compared with samples incubated at 37 °C. The same trend was observed for the aggregation profile, indicating a lower aggregation temperature for the cold stored sample. Both results were consistent with a more compact thermally stable antigen following incubation at 37 °C. Notably, the aggregation temperature was higher for the cold stored samples in the case of ancestral trimer.

The transition between canonical and open trimeric conformation has not been previously reported for Beta spike antigen. Cryo-EM was used to examine recombinant Beta trimers and to follow the effect of cold storage (Extended Data Fig. 2A). The most populated 2D-classes for freshly produced Beta spike antigen correspond to canonical trimeric spike. During cold storage the presence of canonical trimer gradually declines, and no canonical 2D-class species are present after 6 weeks at 4 °C. Our findings indicate that Beta trimer stored for long periods undergoes reversible transitions. Specifically, the spike antigen in the open trimer conformation transitions to a canonical conformation when subjected to incubation at 37 °C. Similarly, when the spike antigen is returned to cold storage, the canonical trimer transitions back to the open trimer conformation (Extended Data Fig. 2B).

### Cryo-EM structure analysis

**Figure 2:**
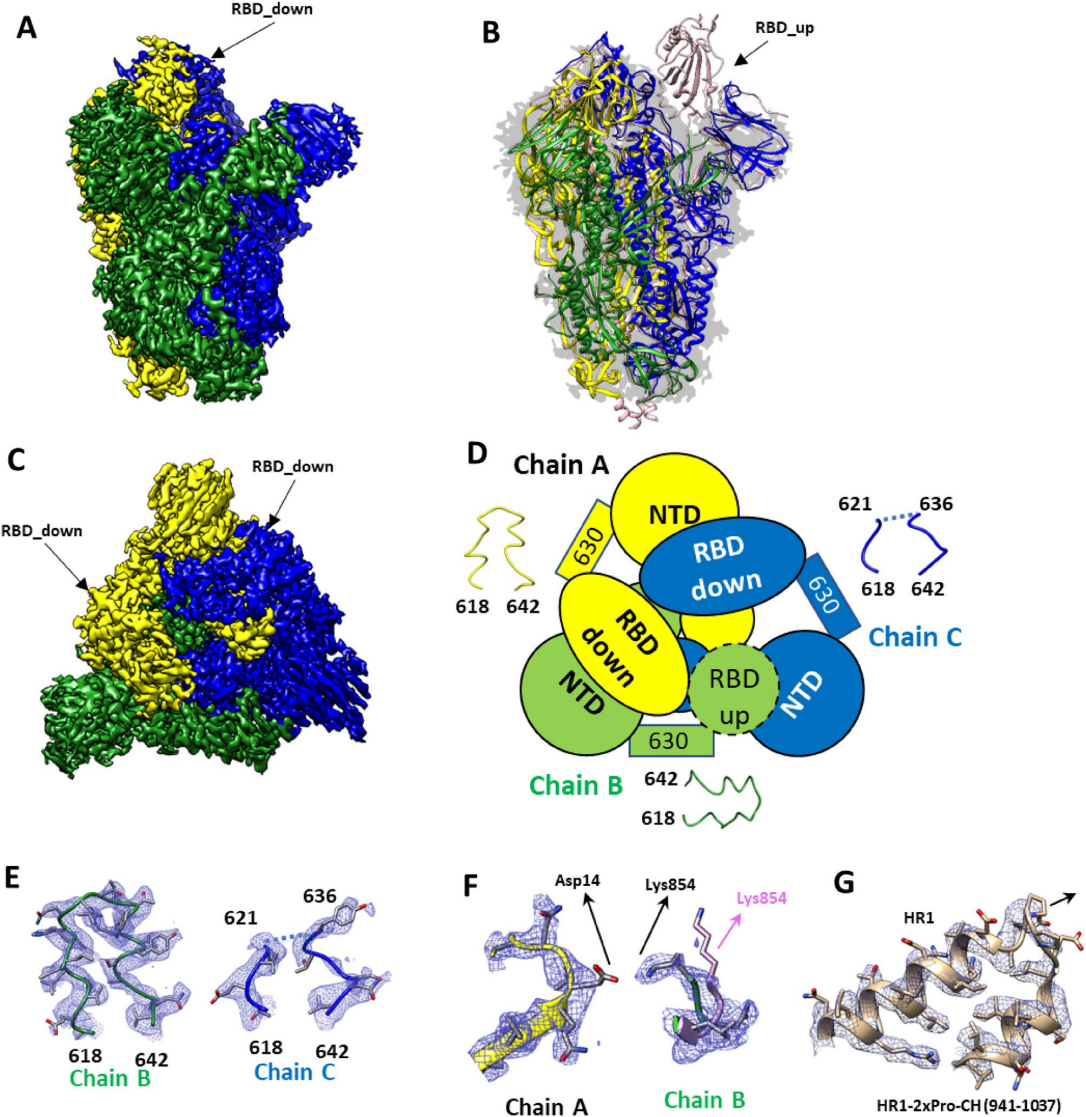
Structural analysis of ancestral trimer. (**A**) Side view of the cryo-EM map of ancestral trimer showing electron density for chain (**A**), (**B**) and (**C**) in yellow, green or blue respectively. RBD domains in the down conformation are visible for chain (**A**) and (**C**). No density for RBD up in chain B is observed at this electron threshold. (**B**) Ancestral trimer model overlap with 6vyb in pink^16^ both fitted in our electron density in light grey. The absence of electron density for the RBD up domain and trimeric helix can be clearly observed. (**C**) Top view of the ancestral spike antigen map. (**D**) Schematic representation of the RBD, NTD, central and 630 loop showing how the orientation of the RBD is associated with the rigidity of the 630 loop region. The RBD up is in dash line since it is only visible at lower electron density threshold. (**E**) Model fit into electron density for the 630 loop region showing the clear density for chain B and the absence of density for most of the loop for chain C. (**F**) Salt bridge between D614 and K854 from chain (**A**) and (**B**) respectively that is absent in 6vyb (pink)^16^. (**G**) CH-HR1 region showing the 2xPro mutation.

**Figure 3:**
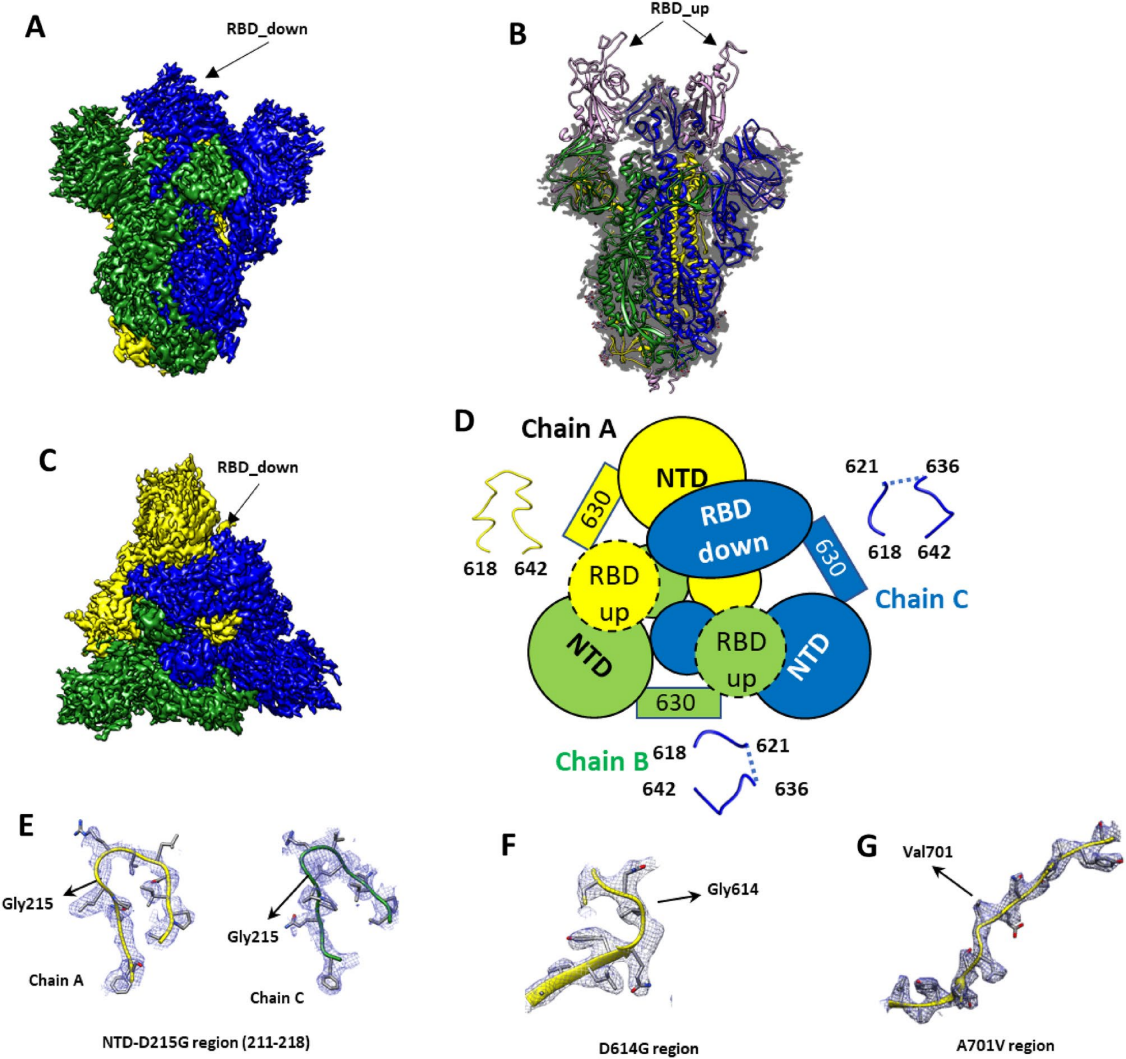
Structural analysis of Beta trimer. (**A**) Side view of the cryo-EM map of Beta trimer showing density for chain (**A**), (**B**) and (**C**) in yellow, green or blue respectively. Only the RBD from chain (**C**) in the down conformation is visible in our map. No density for the RBD up in chain (**A**) or (**B**) is observed at this electron threshold. (**B**) Beta trimer model overlap with 7lyk in pink^21^ both fitted in our electron density in dark grey. The absence of electron density for the RBD up domains and trimeric helix can be clearly observed. (**C**) Top view of the coloured ancestral trimer electron map. (**D**) Schematic representation of the RBD, NTD, central core and 630 loop showing how the orientation of the RBD is associated with the rigidity of the 630 loop region. The dash line for RBD up indicates the lack of density at the selected threshold value. (**E**) Model and electron density showing the region of the D215G mutation for chain (**A**) and (**C**). (**F**) D614G region. (**G**) A701V region.

The cryo-EM structures were determined for both ancestral and Beta spike trimers after incubation at 37 °C. Single particle ab-initio 3D-classification identified one main canonical 3D-class or map for the trimeric protein that corresponds to a single canonical conformation. The ancestral spike trimer map represents the prefusion conformation where two RBDs are in a down conformation, while the third one is facing upward in the up conformation. This quaternary structure is referred to as the one_RBD up conformation and was refined to 2.85 Å (Fig. 2, Supplementary Figs. 2 and 3). The overall architecture of the trimeric antigen is similar to structures reported previously^16,19,20^. As opposed to the ancestral trimer, most Beta trimeric particles are classified into a single model with two RBDs in the up conformation (here referred as two_RBD up conformation). This map was refined to 2.75 Å (Fig. 3, Supplementary Figs. 4 and 5). The overall architecture is very similar to the one observed in Gobeil et al.^21^ (pdb code 7lyk).

Both maps are clearly defined in the S2 region with the local resolution maximum within the core. Consistent with previous findings, no density is observed for the timerization helix (residues 1141–1147). The absence of density for this region that precludes the HR2 and T4 foldon sequences indicates a degree of flexibility within the region (Figs. 2 and 3, Supplementary Figs. 3 and 5). The local resolution gradually reduces when moving from S2 to S1 and reaches its minimum in the distal RBD and NTD domains for both maps. The minimal resolution for ancestral trimer is observed for both-down oriented RBDs. The up-oriented RBDs are only visible when reducing the electron density threshold and no density is observed for the distal part (Supplementary Fig. 3C). Finally, the density corresponding to the 630 loop varies among each protomer and it correlates with the orientation of the neighbouring RBD (Figs. 2D and 3D). Density for the Beta trimer up-oriented RBD is only visible when reducing the electron threshold (Supplementary Fig. 5A and B). As reported for ancestral trimer, the density in the 630 loop is different for each chain.

The 2xPro mutation was clearly visible in both the ancestral and Beta trimer models (Fig. 2G), while the other modifications fall within regions that are not resolved. Orthogonal protein characterization analyses were employed to better understand the differences in conformation transition between ancestral and Beta trimers.

### Surface plasmon resonance (SPR)

**Figure 4:**
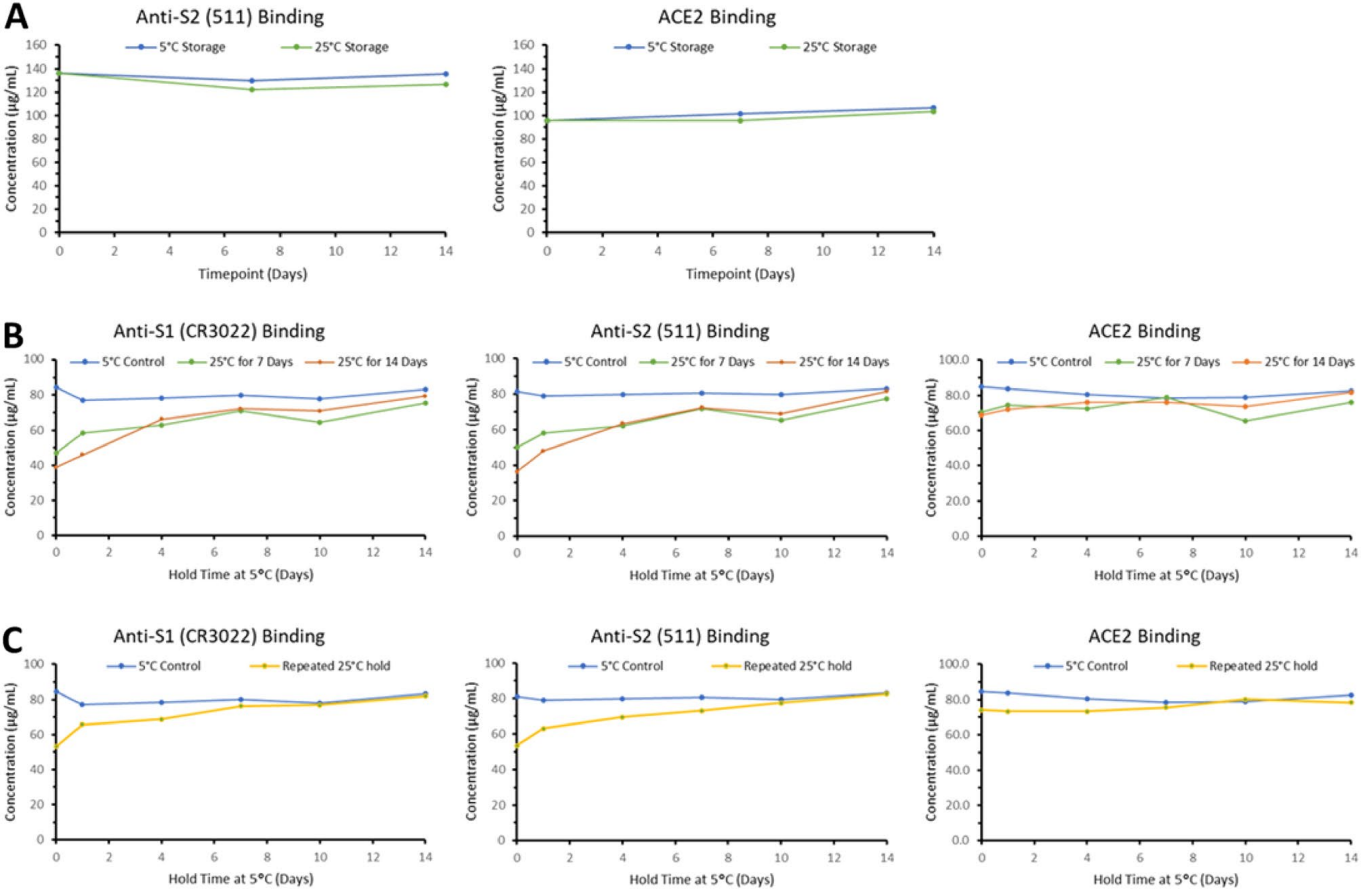
SPR analysis. Trimer temperature conformation effect after thermal stress (25 °C) using anti-S1, anti-S2 and ACE2 binding. (**A**) Ancestral trimer showing no loss of anti-S2 or ACE2 binding after thermal stress for 14 days. (**B**) Recovery of anti-S1 and S2 binding when Beta trimer is held at 5 °C for up to 14 days after thermal stress for 7 & 14 days. ACE2 binding was not affected. (**C**) Similar binding recovery observed for Beta trimer after repeated 25 °C-5°C-25 °C-5°C treatment.

To map epitope availability through simultaneous real-time concentration determination, spike antigen binding to ACE2 receptor and antibodies recognizing the RBD (CR3022) and S2 (511) domains was determined using SPR (Table 1). Thermal stress of ancestral trimer at 25 °C did not result in any significant loss of anti-S2 or ACE2 binding with continuous storage up to 14 days (Fig. 4A). When Beta spike antigen was subjected to short-term thermal stress for 7 to 14 days and restored to 4 °C storage, a 50% loss of initial binding to the CR3022 and anti-S2 epitopes was observed. With subsequent 4 °C storage, binding to these epitopes was restored within 14 days. However, binding to the ACE2 receptor was less affected (Fig. 4B), suggesting that the receptor binding site remains available when transitioning from canonical to open trimer conformation. Repeated 25 °C-4 °C-25 °C-4 °C treatment (Fig. 4C) demonstrated that this conformational transition is entirely reversible without structural changes under these conditions.

**Table 1:** Spike trimer binding sites for ACE2 and antibodies.

Binding partner	Ancestral	Beta
511	969-988 (SRLDPPEAEVQIDRLITGRL)	966-985 (SRLDPPEAEVQIDRLITGRL)
*CR3022	356-379 (YNSASFSTFKCYGVSPTKLNDLCF)	353-376 (YNSASFSTFKCYGVSPTKLNDLCF)
	414-417 (DDFT)	411-414 (DDFT)
	502-505 (FELL)	499-502 (FELL)
**hACE2	426 (N), 436 (Y), 440(Y), 442-3 (LF), 462 (A), 473-4 (FN), 476 (Y), 480 (Q), 482 (Y), 485 (Q), 487-9 (TNG), 492 (Y)	423 (N), 433 (Y), 437(Y), 439-440 (LF), 459 (A), 470-1 (FN), 473 (Y), 477 (Q), 479 (Y), 482 (Q), 484-6 (TYG), 489 (Y)

*CR3022 epitopes^22^.

**hACE2 binding site^22^.

### High-resolution SEC-UPLC experiments

**Figure 5:**
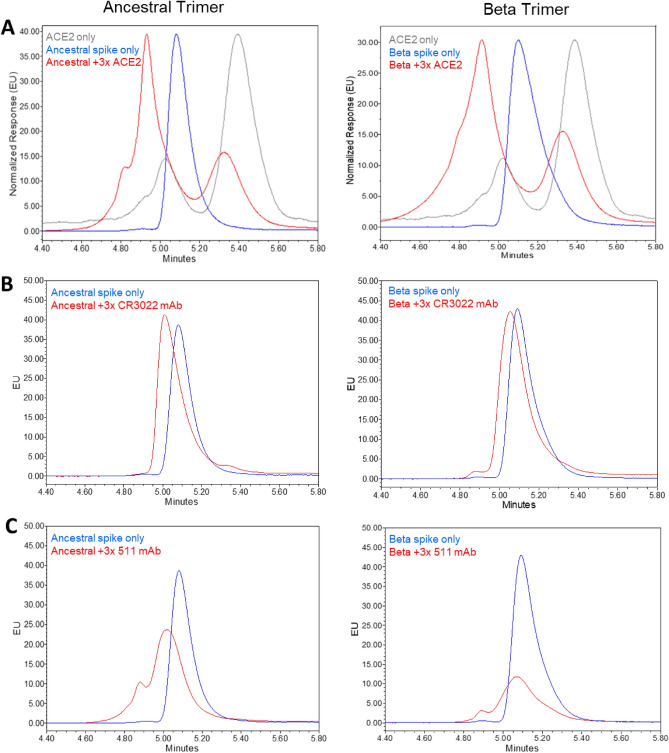
High-resolution SEC-UPLC analysis. Ancestral and Beta trimer binding to (**A**) ACE2 receptor, (**B**) anti-RBD CR3022 antibody, (**C**) anti-S2 511 antibody. While saturation at 3 × molar excess antibody was observed for ancestral trimer (shift in retention time and peak shape), incubation of Beta trimer with the same anti-S2 511 antibody did not result in similar shifts in the chromatographic profile even up to a 12 × molar excess (data not shown). All conditions were held at 2 to 8 °C for SEC-UPLC analysis. Response of ACE2 only control trace (grey) was normalized to spike protein only (blue) to allow for visualization. The recombinant ACE2 elutes as two separate peaks with the homodimer eluting at approximately 5.4 min and the dimer of homodimer eluting earlier at 5.0 min.

Using SEC-UPLC, we observed no differential binding of either trimer when incubated with increasing concentrations of ACE2 or anti-RBD antibody (CR3022) whose binding epitopes have previously been characterized^22^ (Fig. 5A and B). The uniform recognition of antibody CR3022 by SEC-UPLC for both trimers is consistent with the highly conserved epitope reported for this cross-neutralizing antibody^22^ and with the RBD conformational positioning observed for ACE2 binding. However, there was a clear change observed in the binding properties when incubating ancestral trimer with increasing concentrations of anti-S2 antibody (511) compared to Beta trimer, which showed no change (Fig. 5C). While saturation at 3 × molar excess antibody was observed for ancestral trimer, incubation of Beta trimer with the same anti-S2 antibody did not result in saturation. The lesser extent of anti-S2 antibody binding for Beta compared to ancestral trimer can be attributed to the observation of the differential RBD position in the canonical conformation in which anti-S2 antibody (511) is unable to bind to spike antigen, however this was unexpected for antigen in the open trimer conformation. To investigate why S2 availability appears to be different for Beta trimer, additional experiments were performed to better understand the open trimer transition to canonical conformation during incubation at 37 °C.

**Figure 6:**
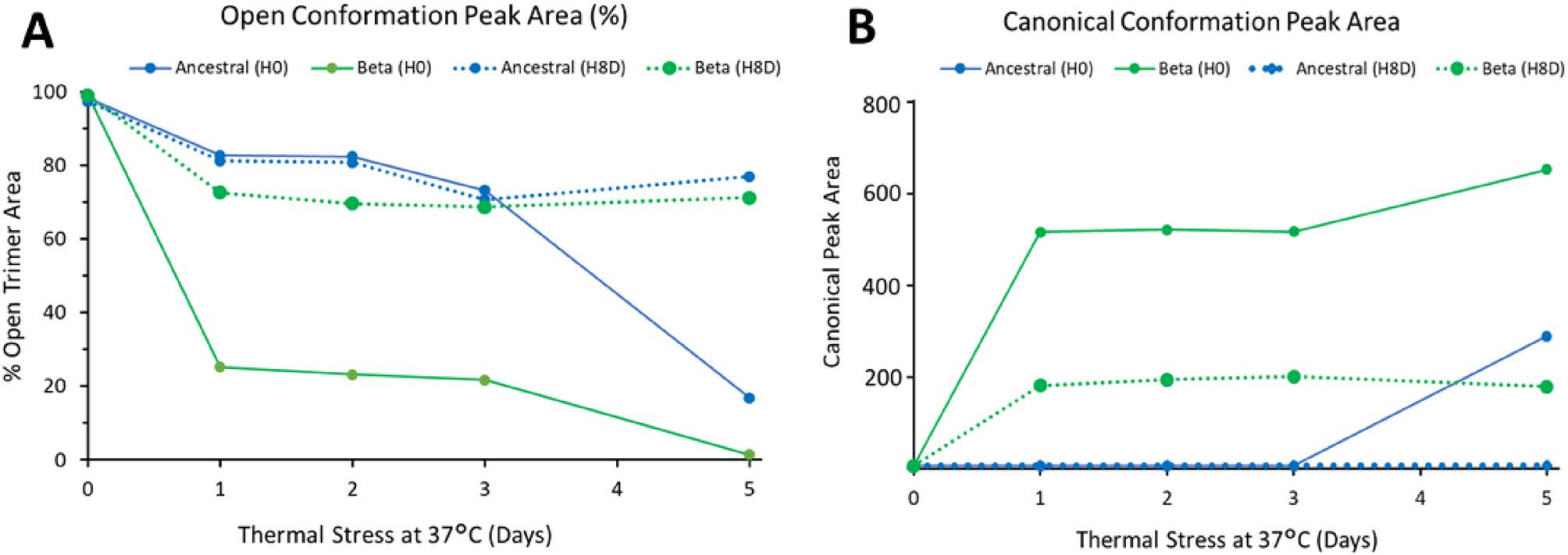
SEC-UPLC analysis showing conformational transition after thermal stress (37 °C) and cold storage for 8 days. (**A**) Percent open trimer peak area for ancestral and Beta spike protein yielding partial recovery of open trimer conformation after temperature treatment followed by cold temperature hold. (**B**) Canonical trimer peak area comparison for ancestral and Beta spike antigen in which Beta transitions more rapidly from open trimer to canonical conformation compared to ancestral trimer. H8D denotes 8 day hold at cold storage whereas H0 represents no hold after thermal stress.

Both ancestral and Beta trimers were incubated at 37 °C and SEC-UPLC elution times were compared to measure the amount of open trimer and canonical conformation during incubation at 37 °C. Unexpectedly, a difference in both the rate and completeness of conformational transition from open trimer to canonical was observed. Beta spike trimers transitioned from 100% open trimer to ~ 80% canonical trimer in one day after incubation at 37 °C and by day 5 were all in the canonical state. Ancestral spike trimers transition at a slower rate taking three days to shift meaningfully to the canonical conformation with only ~ 80% canonical conformation after the 5-day time point (Fig. 6A). Interestingly, upon returning to 2–8 °C for 8 days after the 37 °C incubation, the opposite was observed with ancestral spike trimer reverting nearly completely from canonical to open trimer conformation within one day and the Beta spike trimer reverting more slowly and incompletely (Fig. 6B). To investigate the contribution of the S2 domain in this structural transition, Hydrogen–Deuterium Exchange by Mass Spectrometry (HDX-MS) was undertaken.

### HDX-MS analysis of the Beta trimer

**Figure 7:**
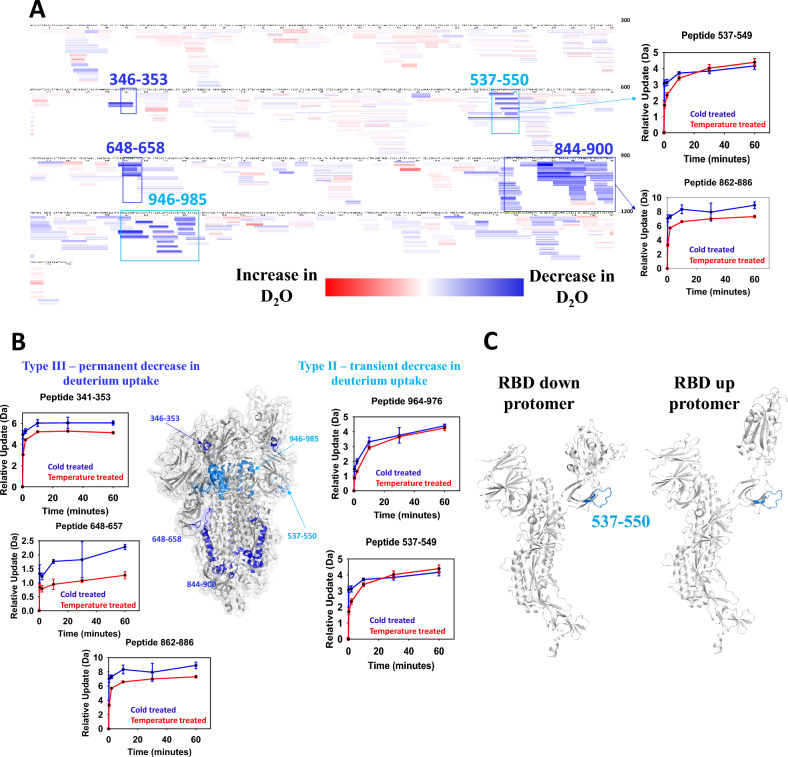
HDX-MS analysis. (**A**) Dynamic analysis of temperature treated Beta trimer at 37 °C for 24 h: Heat map analysis of Beta spike antigen comparing cold stored vs temperature treated. Sequence coverage resulting from HDX-MS was 93.4%. The magnitude of difference is demonstrated in a red-blue spectrum format. Red and blue represent increase and decrease in deuterium uptake for temperature treated sample respectively. Type II exchange peptides are highlighted in cyan whereas Type III exchange peptides are highlighted in blue. (**B**) Dynamic analysis mapped onto 3D structure. The HDX data for temperature treated Beta spike antigen mapped onto representative 3D structure (PDB: 7LYQ). Type II exchange regions are highlighted in cyan. Type III exchange regions are highlighted in blue. The kinetic plot of representative peptides in those regions are also demonstrated and colour coded accordingly. In the kinetic plot, cold treated sample is presented in blue whereas heat treated sample is shown in red. (**C**) Close look at residues 537–550: RBD down conformation is shown on the left and RBD up conformation is shown on the right. Type II exchange region (residues 537–550) is shown in cyan. For simplicity, only one protomer is shown.

HDX-MS was performed to compare the conformational dynamics of the Beta trimer between the cold storage and 37 °C incubated forms. HDX-MS is used to measure dynamic changes in the hydrogen bonding network through hydrogen/deuterium exchange across amide bonds of the primary sequence of a protein system. The technique does not resolve down to individual amino acid residues, but instead uses protease-based digestion to generate an overlapping sequence coverage map. Sequence coverage of the antigens for these experiments was ~ 94%. Most of the missing sequence information was observed around N-glycosylation sites (Fig. 7A). N-linked glycans can restrict protease access adjacent to the modification site which decreases digestion efficiency and can result in reduced local sequence detection in these regions.

When the Beta trimer sample incubated at 37 °C was compared to the cold stored antigen, differences in deuterium exchange were observed in both the S1 and S2 domains. Regions of dynamics exchange detected in the 37 °C incubated sample included sequences 346–353 and 537–550 of the S1 domain and 648–658, 844–900, and 946–985 regions of the S2 domain (Fig. 7A and B). Upon further analysis of the deuterium uptake kinetic plots, two types of HDX behaviour were noted: Type II and Type III (Fig. 7A and B)^23^. Type II kinetics are indicative of changes in dynamics, where the exchange rates are affected, but the total conformational freedom in a specified sequence is maintained across both conformations of the Beta trimer structure. Hence Type II behaviour can be thought of as transient changes within the experimental mixing timescale that are able to sample same state as cold stored Beta trimer. Type III kinetics show a change in protein structure that can result from alterations to the secondary structure or a change in folding, tertiary structure, that alters amide site exposure. Changes are permanent in the timescale that is measured (e.g. a new H-bond is formed, or an H-bond is broken). Dynamics changes (Type II) were only detected in the head region of the antigen, S1 and part of S2 domain, while Structural changes (Type III) were primarily localized to the stalk region of the Beta trimer, S2 domain (Fig. 7B). For example, representative peptides 964–976 and 537–549 exhibited Type II exchanges, where deuterium uptake was observed at earlier mixing timepoints (20 s, 2 min, and 10 min, Fig. 7B). However, at later mixing timepoints (30 and 60 min) the differences were negligible. Representative peptides 341–353, 648–657, and 862–886 exhibited Type III kinetics. Changes were permanent in the timescale measured and different final levels of deuterium uptake were observed at later timepoints.

Overall, a global decrease in deuterium exchange was observed in the analysis of 37 °C incubated compared to cold stored samples. This decrease can be interpreted as a shift to a more compact conformation in the 37 °C incubated state. These results are consistent with structural observations in cryo-EM, SEC, and nanoDSF analysis.

## Discussion

Antigen structure and its presentation to the immune system is critical to the development of a protective immune response upon vaccination. In this work we present the cryo-EM elucidated structure of the Sanofi Beta trimer and apply SPR, SEC, nanoDSF, and HDX-MS for conformational analyses to better understand how the unique structure, dynamics, and stabilizing features of this antigen lead to improved overall vaccine performance. The T4 foldon stabilized structure induced superior overall stability of the spike trimer in a temperature and epitope-dependent manner. This was confirmed in the SPR and SEC experiments using different molecular probes to compare subtle structural differences between the two spike antigens. The primary aim of this study is to provide a more rational understanding of the superior, broadly cross-neutralizing, antibody response elicited after administration of the Beta spike antigen booster vaccine^10–13^. Our studies provide insights into the structural and biological properties of this antigen which demonstrate stability and binding selectivity in a temperature-controlled manner. We conclude that the modified structural moieties of the Beta antigen versus the ancestral trimer likely combine for the superior cross-neutralization immunogenicity observed in the clinical data^12,13^.

### Canonical vs open-trimeric spike antigen

Our results show that both antigens stored at 4 °C remain trimeric and capable of transition from the preferred cold stored open trimer conformation to a canonical conformation observed at physiologic temperature. Cryo-EM single particle analysis shows an abundance of canonical folded particles gradually transition to the open trimer form after cold storage. This cold storage sensitivity was previously reported for a very similar ancestral spike construct and was postulated to be more pronounced for spike antigens where the RBD is in an up conformation^18^. Our work demonstrates for the first time that this cold storage sensitivity also affects Beta spike antigen. We also show that following cold storage, the protein remains trimeric, and the process is reversible after additional heat/cool cycles, indicating the existence of an alternate conformation for cold stored antigens. This alternate conformation is visible on the cryo-EM grids but does not form defined 2D-classes, which together with our HDX, SEC-UPLC, and nanoDSF results indicate a higher flexibility and heterogeneity of this alternate conformation.

### Structural differences between spike antigens

Our structural work showed a canonical form for the ancestral trimer that is characterized by one RBD in the up conformation. These findings coincide with the structure described by Wrapp et al.^16^. Some reports observed that similar protein constructs exist in an equilibrium between the one_RBD up and down conformation, while other reports describe the ancestral trimer in the fully down conformation^24,25^. The conformational discrepancy between the RBD position in these similar constructs remains a topic of debate and has been attributed to multiple factors (sample incubation temperature, excipients, and purity).

For the Beta trimers, particles can be classified into a unique 3D model where most of the population exhibited two RBDs facing up (two_RBD up conformation). This structure is similar to one previously described for Beta trimers^21^ (pdb-code:7lyk). However, in that report the trimer was more heterogeneous showing six different quaternary structures: two closed conformation, four one_RDB up conformations and finally the two_RBD up conformations, with each group representing 15%, 72% and 13% of the particles respectively. This heterogeneity was lower in other reports where the Beta trimer was found to be in a one_RBD up conformation and a transition state representing each half of the particles^26^. Remarkably, all reports identify higher percentages of the RBD up state for the Beta trimer when compared to ancestral trimer. Moreover, this fusion prone tendency observed for Beta is more pronounced in our structure, where only the two_RBD up conformation is present. The two_RBD up conformation of the Beta trimer stochastically presents the three RBD amino acid substitutions, K417N, E484A, and N501Y present in most of the VOC including Omicron, resulting in the potential for higher elicited cross-neutralizing antibody titers^27^.

No clear density for the trimeric alpha helix (residues 1141–1147) was observed in either spike antigen structure. The absence of density for the C-terminal region indicates that the T4 foldon is located in a part of the trimer with a high degree of flexibility as previously reported^16,19–21^. This flexibility is also present within the SARS-CoV-2 virion particle, where the region between the transmembrane domain and the 1141–1147 helix acts as a flexible linker that allows the spike protein to adopt different orientations^28^. This flexibility could be the key attribute that facilitates interconversion between open trimer/canonical conformations while maintaining T4 foldon stability and thus keeping the trimer together. The HDX-MS results did not detect significant differences at the C-terminal T4 foldon region between canonical and open trimer states (deuterium uptake difference was negligible between cold stored, and 37 °C incubated samples) indicating a consistent structure in both conformations. SEC results also show that the trimer structure is maintained between cold-stored (open trimer) and 37 °C incubated (canonical) conditions.

In all the above experiments, we have observed conformational changes between cold-stored and incubation of samples at 25 °C or 37 °C. HDX-MS analysis was performed to identify regions of the Beta spike primary sequence where (i) there was a difference in the conformational dynamics or (ii) protein structure has changed. In the S1 domain, residues 537–550 are situated near the base of the RBD. These residues exhibited type II behaviour implying that the RBD had similar flexibility to assume up- or down- conformations, but that the equilibration kinetics decreased compared to the open trimer structure (Fig. 7B and C same maximum uptake). There was also a structural change, detected as Type III kinetics, that mapped onto a section of helix in the RBD. This suggests that the helix in this region of the RBD is stabilized in the canonical timer. We also note that deuterium exchange rates in the ACE2 binding motif were unchanged by the incubation at 37 °C.

The Beta trimer adopted a more compact structure as shown by SEC-UPLC data and affected areas were mainly localized in the S2 domain consistent with Costello et al.^17^. From the HDX-MS analysis, the helices close to the ectodomain (residues 946–985) were able to slowly transition between open and close conformation within the dynamics mixing timescale while internal helices (residues 648–658 and 844–900) in the S2 domain were more rigid upon incubation at 37 °C (Fig. 7B). While the affected residues in the Beta trimer S2 domains are similar to those reported for ancestral trimer^17^, our study extends the D614G substitution impact to conformational structure observation^29^ to identify different kinetic behaviours between the two spike antigens based on amino acid substitutions in the Beta variant S2 domain. We observed a clear change when incubating ancestral trimer with anti-S2 antibody (511) compared to the Beta trimer, which showed no change. The 511 epitope overlaps with residues 946–985, helices that are shown to transition between open trimer and canonical conformations. The SEC-UPLC data demonstrated that Beta trimer transitions from open trimer to canonical state faster than ancestral trimer and the SPR data showed selective binding in the S2 region of the Beta trimer which could be contributed to the antigen sampling multiple conformations (Figs. 4 and 6). Thus, the binding of Beta trimer is temperature and epitope dependent. For vaccines stored routinely at 4 °C, the protein is stable in the open trimer conformation in a receptor ‘up’ conformation and the S2 domain is available for efficient binding. We believe this contributes to the rapid transition to the canonical conformation and slower transition back to an open trimer conformation for the Beta trimer allowing the two_up canonical state to remain in place at physiologic temperature.

The conformation of the spike trimer has a direct impact on SARS-CoV-2 transmission and immune evasion by positioning the RBD of each protomer in either an up or down state. The primary driver of conformational change is sequence variability in the SARS-CoV-2 spike antigen domains, which affects conformational stability as well as a playing a direct role in varying the neutralizing antibody epitope presentation^30^. Secondary mechanisms for vaccine recombinant protein conformational stability include the engineered T4 foldon trimerization domain and post-translational glycosylation provided by the baculovirus expression system^31^ (both ancestral and Beta spike trimers used the same T4 foldon sequence and found to have similar glycosylation patterns). Here we demonstrate the Beta spike trimer in the Sanofi COVID booster vaccine has a preferential two RBD up conformation, providing twice the surface area of exposed RBD amino acids compared to the ancestral spike trimer booster vaccine. Three key mutations in the Beta spike RBD (K417N, E484A, and N501Y) are the target for two thirds of the most potent neutralizing antibodies identified from Beta SARS-CoV-2 infected individuals^32^, thus contributing to the broadly cross neutralizing immunogenicity demonstrated in the clinical development of the vaccine^12,13^ and the real-world effectiveness against Omicron XBB.1.5^33^.

## Conclusion

Overall, we found that both trimers are stable, homogeneous at constant temperature, and configured in the antigenically preferred prefusion conformation with a fusion prone RBD orientation (one or two_RBD up), consistent with the literature reporting spike antigen structures. We report notable differences in the rearrangement of RBD domains opening the door to a better understanding of differences in vaccine efficacies. However, after incubation at 37 °C, cryo-EM results show that both of our antigens have a single quaternary structure. This homogeneity and consistent binding epitope availability in our vaccine antigens could reflect a more reproducible immunogenic response. Unlike the structures reported for other recombinant spike trimers, where the protein is in a closed canonical conformation with all RBD down^25^, both Sanofi vaccine antigens are in an RBD-up conformation potentially allowing the display of a broader antigenic surface. This is particularly important for the Beta trimer where the antigen shows a canonical quaternary structure with two RBD in the up position and is consistent with the similar clinical effectiveness observed in the UK Spring vaccine campaign utilizing both Sanofi Vidprevtyn Beta and Pfizer Omicron bivalent booster vaccines^32,33^.

The kinetics for the temperature transition observed between the canonical and open-trimer conformation has been shown to be different for VOC^17^. This transition not only increases the antigenic exposure of the RBD but also reveals highly conserved regions that could act as potential epitopes for pan-coronavirus antibodies. This could contribute to the broad protective nature of our Beta booster vaccine as demonstrated by the elicited broad cross neutralization^10,13^ and functional antibody responses (Fc-γ-R binding and IgA)^34^ against all tested variants of concern.

## Methods

### Generation of the SARS-CoV-2 recombinant prefusion S protein

Recombinant prefusion spike protein was produced from a Sanofi proprietary cell culture technology based on the insect cell baculovirus expression vector system. The sequence was based on the Wuhan YP_009724390.1 strain spike sequence, modified to improve the conformation, stability, and trimerization and to facilitate the purification. The modifications comprised the introduction of two proline mutations in the C-terminal region of S2 domain, which was previously shown to stabilize the protein in a prefusion conformation. Briefly, the modified sequence was cloned into a baculovirus transfer plasmid, which was then used to generate a recombinant baculovirus containing the gene of interest. The recombinant baculovirus was first amplified in expresSF + insect cells prior to infecting a large scale expresSF + insect cells culture in suspension. After incubation, the recombinant protein was purified from the supernatant using several affinity and chromatography columns.

Purity was determined by SDS-PAGE, scanning densitometry and reversed-phase liquid chromatography (RP-HPLC). Spike protein identification was confirmed by Western Blot using affinity purified rabbit polyclonal antisera specific for the spike receptor binding domain (RBD) expressed in HEK293 cells with only one band of approximate MW of 160 kDa detected, corresponding to the full-length spike protein. The purity of this material was determined to be > 90% by SDS-PAGE using densitometry. Two orthogonal methods were used to verify the accuracy of the purity measurement. Analysis by RP-HPLC showed one major protein peak with minor leading and trailing peaks which were determined to be spike protein-related based on fractionation followed by mass spectrometry (MS) and Simple Wes (automated Western Blot) analysis. The resulting purity by RP-HPLC was > 90%. In-solution digestion of the whole sample followed by LC–MS/MS sequence identification of proteolytic peptides also yielded results consistent with highly purified protein antigen.

### Temperature effect analysis by nano DSF and SEC

Duplicate thermal shift assays were conducted for both cold stored and 37 °C incubated antigens by nano Differential Scanning Fluorimetry (nano DSF) based on intrinsic protein fluorescent signal using the Prometheus NT.48 nano DSF instrument (NanoTemper Technologies GmbH, München, Germany) equipped with manufacturer-specified capillaries. In these assays, the sample solutions were subjected to a heating rate of 1.5 °C/min, with temperature ranging from 20 °C to 95 °C. Fluorescence measurements were recorded at 330 nm and 350 nm, with excitation performed at 280 nm. The fluorescence ratio between the detected signals at 330 nm and 350 nm was plotted against temperature, allowing the determination of the melting temperature at the inflection point of the resulting sigmoidal curve. Additionally, scattering curves depicting thermal unfolding (up) and its first derivative were included for analysis.

Size exclusion chromatography (SEC) experiments were performed in an Agilent 1260 Infinity II (Agilent Technologies, United States) analytical HPLC system with a UV–Vis detector using a Superose-6 3.32/300 column (GE Healthcare). Ancestral and Beta spike antigens were concentrated to 3 mg/mL and stored at 4 °C for several weeks (cold stored antigen). An aliquot was incubated at 37 °C for one day (temperature treated antigen). Finally, cold stored and temperature treated samples were loaded onto the SEC column and the obtained chromatograms were superposed by scaling each chromatogram to the highest peak.

### Cryo-EM sample preparation and data collection

The ancestral and Beta spike antigen samples were each concentrated to 3 mg/mL. Samples were then incubated for 1 day at 37 °C. For grid preparation, 3.5μL of protein was applied onto a 200 Mesh Ultrafoil Grid. Grid vitrification was carried out using a Vitrobot-IV apparatus from Thermo Fisher Scientific, with a humidity level of 100%, ashless filter paper (Standard Vitrobot Filter Paper, Ø55/20 mm, Grade 595; Electron Microscopy Sciences), a blot time of 4 s, and a blotting force of 4. The grids were then stored in liquid nitrogen and subsequently collected using a 200 keV Thermo Fisher Scientific Glacios Cryo-Transmission Electron Microscope equipped with a Falcon 4 direct electron detector camera. Image acquisition was performed using EPU software 2.9 (Thermo Fisher Scientific) with a defocus range between -0.8 and -2.2 μm. The pixel size was set to 0.58 Å (Table 2). Each movie consisted of 55 frames, with an overall dose of 62 e/Å2. A total of 19,667 movies were collected for ancestral, while 10,494 movies were collected for B.1.351 spike antigen.

**Table 2:** Cryo-EM data collection, refinement, and validation statistics.

	Ancestral	Beta
Data collection and processing		
Grid type	UltraAuFoil 200 mesh R1.2/1.3	UltraAuFoil 200 mesh R1.2/1.3
Plunge freezer	Vitrobot	Vitrobot
Magnification	240,000	240,000
Voltage (kV)	200	200
Camera	Falcom4	Falcom4
Electron exposure (e/Å^2)	62	62
Defocus range (µm)	(− 0.8 to − 2.2)	(− 0.8 to − 2.2)
Pixel size (Å)	0.58	0.58
Micrographs	19,667	10,494
No. of fractions	55	55
Symmetry imposed	none	none
Initial particles imaged (no.)	2,618,182	2,186,869
Initial particles imaged (no.)	349,329	262,895
Map resolution (Å)^#^	2.85	2.75
FSC treshold	0.143	0.143
Refinement*		
Model resolution (Å)^#^	3.1	3
FSC treshold	0.5	0.5
Model resolution range (Å)		
Map sharpening *B* factor (Å^2)	93.9	79.5
Model composition		
Non-hydrogen atoms	20,801	19,482
Protein residues	2659	2454
Ligands	35	29
* B* factors (Å^2)		
Protein residues (mean)	76.81	71.77
Ligands (mean)	100.06	90.52
R.m.s deviations		
Bond lenghts (Å)	0.002	0.003
Bond angles (º)	0.455	0.478
Validation		
MolProbity score	1.4	1.54
Clashscore	4.77	4.69
Ramachandran plot		
Favored (%)	97.18	95.73
Allowed (%)	2.82	4.27
Disallowed	0	0

^#^resolution from Cryosparc v3.2.0.

*Using comprehensive validation in Phenix (1.20.1–4487).

### Data processing

Raw micrographs were imported into Cryosparc^35^. Movie frames were aligned using patch motion correction and contrast transfer function (CTF) estimation was performed using Gctf^36^. Using the curate exposure feature all micrographs with CTF fit resolution lower than 5 Å were excluded. Blob picking was run, including elliptical blob option, using 90 and 210 Å as minimum and maximum particle diameter respectively. Particles were extracted using a down sampling factor of 2. The first good particle dataset was selected by three consecutive runs of Ab-initio 3D classification into three classes (Supplementary Figs. 2 and 4). This good particle dataset was further cleaned by 5 runs of heterogeneous refinement. Finally, a non-uniform refinement followed by homogeneous refinement was performed^37^. The selected particles were re-extracted without down sampling and after a round of local CTF refinement followed by homogeneous refinement the final map was obtained^38^.

The cryo-EM structure of ancestral spike antigen with a unique RBD up (pdb code:6vyb) was used as the starting point for building the ancestral spike model. A second cryo-EM structure with two RBD up (pdb code:7lyk) was used for building Beta spike antigen. Model visualization and first fitting into cryo-EM maps was performed using the programs UCSF Chimera^39^. An initial manual model building was performed in coot^40^. The structure was then refined through iterative rounds of manual model building and real-space refinement in Phenix^41^. The structure was validated using MolProbity^42^.

### Cryo-EM 2D-classes

A freshly prepared sample of Beta spike antigen was concentrated to 3 mg/mL and then incubated at 4°Celsius. Every two weeks, an aliquot was taken from the sample and grids were frozen and stored in liquid nitrogen. After more than 6 weeks of cold storage, the sample was transferred to 37°Celsius for one day. Subsequently, the sample was once again incubated at 4°Celsius for several weeks, and grids were frozen every two weeks during this period.

For each grid, triplicate preparations were made. After collecting a small dataset consisting of 500 micrographs, a 2D classification was performed to screen for the abundance of canonical spike particles.

### SPR

Surface plasmon resonance (SPR) was performed using a Cytiva Biacore T100. A Protein A/G surface was prepared through amine coupling of Protein A/G (Pierce) onto a CM5 Series S sensor chip (Cytiva) at pH 4.5 for 10 min. ACE2-Fc-Avi-His (human ACE2, Sydlabs), anti S2 (511) (AbCellera, Inc) and anti-RBD (CR3022) (Fisher) ligands were captured onto the Protein A/G surface at a flow rate of 10 µL/min for 30 s. Antigen samples were serially diluted in 1X HBS-EP + running buffer and injected over the surface of the sensor chip at a flow rate of 30 µL/min for 60 s, and the interaction response between analyte and ligand was measured as a function of the change in the refractive index of the solution, which is directly proportional to the mass of the bound analyte. Regeneration was performed using 50 mM sodium hydroxide at a flow rate of 10 µL/min for 10 s. The measurement was performed to determine analyte concentration was determined against a 6-point calibration curve generated from Beta spike reference antigen (Sanofi in house) of known concentration using Biacore T100 Software v2.0.4 (Cytiva).

### SEC-UPLC

High-resolution size exclusion chromatography (SEC) experiments were performed on a Waters H-Class ultra-performance liquid chromatography (UPLC) with ultraviolet and fluorescence detection. The high-pressure capabilities of UPLC facilitated use of sub-2-μm particles for monitoring different conformational forms of higher order structure after thermal stress or incubation with mAb/ACE2 receptor.

Purified ancestral and Beta spike antigen were titrated with 3, 6, 9 and 12 × molar excess of ACE2 receptor, anti-S2 (511) or anti-RBD (CR3022) monoclonal antibodies at 2 to 8 °C. Each sample was analysed in duplicate on an Agilent AdvanceBio SEC 200A 1.9 µm 4.6 × 300 mm column held at 30 °C. Separation was performed using 10 mM NaP 300 mM NaCl pH 7 running buffer with a flowrate of 0.35 mL/min. Chromatograms were collected UV 215 nm/FLD ex. 280 nm, 340 nm and viewed in Waters Empower software.

Heat-treated samples were generated by incubating antigen formulated with 0.2% Tween 20 in PBS, pH 7.0 at 37 °C ± 2 °C for 1 to 5 days or 25 °C ± 2 °C for 0 to 14 days followed by holding at the normal storage condition of 5 °C ± 3 °C for 0 to 14 days. Additionally, one condition involved multiple cycles of temperature stress with a 7-day incubation 25 °C ± 2 °C for 7 days followed by 14 days holding at 5 °C ± 3 °C then re-stressing at 25 °C ± 2 °C for another 7 days then holding samples at 5 °C ± 3 °C for 0 to 15 days. Samples were tested by SPR and SEC-UPLC to characterize reversibility of the conformational change from open trimer to canonical spike. The SEC-UPLC analysis on heat-treated samples was performed using the same methodology described above for mAb/ACE2-incubated samples. Thermal stress yielded a retention time shift to a later eluting population by SEC-UPLC which integrated and area under the curve reported as canonical peak area. The proportion of open trimer was expressed as a percentage of total integrated area and plotted as a quantitative representation of the conformation transition observed by SEC-UPLC.

### HDX-MS

Dynamic analysis of cold stored versus 37 °C incubation. The HDX-MS experiments were conducted as previously described with minor modifications^43^. Non-deuterated control buffer, Buffer E, (10 mM phosphate buffer, 150 mM NaCl with 5% acetonitrile at pH 7.0) was used for digestion controls whereas for deuteration, deuterated buffer E was used with pH of 7.0. Temperature treated samples were generated by incubating Beta spike antigen final product at 37 °C for 24 h. The sample formulation buffer contained a high level of detergent which was not MS friendly. As such, cold stored and 37 °C incubated samples were buffer exchanged extensively in buffer E prior to HDX-MS experiments.

For both states (cold stored and 37 °C incubated), five HDX mixing timepoints (20 s, 2 min, 10, min, 30 min, and 60 min) were acquired in technical triplicates. To ensure consistent comparison, T_0_ sample was stored in 4 °C whereas heat treated sample was stored at 25 °C chamber for the entire sample acquisition (note: 25 °C was the highest temperature limit for the protein storage chamber hence it was used for heat treated sample). The deuterated samples were quenched followed by injection into nanoACQUITY UPLC HDX-MS module containing (1:1) pepsin/protease XIII (NovaBioAssays, MA, USA) for digestion. The digested peptides were subsequently desalted (100 µL/min for 3 min) and separated using Waters BEH C18 guard column (30 µL/min using 7 min gradient) and ACQUITY CSH C18 analytical column respectively. The eluted peptides were detected using Waters Synapt G2-Si mass spectrometer using acquisition of mass/charge (m/z) range between 300 and 1700. GluFib (785.8426 m/z) was used as a lock mass solution to maintain mass calibration of < 10 ppm.

### Epitope screening of mAb 511

Epitope screening was conducted on the ancestral strain. For antigen/antibody complex, equimolar of 1.1 µM was mixed at room temperature for an hour. For HDX experiments, similar buffers were used as above minus the acetonitrile in the digestion or deuteration buffers. Two mixing timepoints were acquired: 10 and 30 minutes^43^ and the LC–MS conditions were similar as above except that the peptides were separated at 40 µL/min using 7 min gradient and the eluting peptides were sprayed using conventional high-flow source instead of ionKey. Significance criteria for epitope determination were adopted from Zhu et al.^44^ where the differences must surpass 0.5 Da and a 3 × standard deviation threshold.

### Peptide identification and deuterium uptake analysis

ProteinLynx Global Server (Waters Corp., MA, USA) was used for peptide identification and DynamX software (Waters Corp., MA, USA) was used for analysis of deuterium exchange on identified peptides. Only peptides with high product per amino acid score and spectral quality were considered. The summed difference over all timepoints were used for comparison evaluations.

Back-Exchange correction factor was not applied in this study as comparison of same peptides were made for different protein states. However, based on the previous publication and study^45^, the back-exchange was calculated to be on average of 29.9% using 11 enolase peptides. In adherence to the recommended guideline for HDX-MS experiment reporting, the raw HDX-MS data for both states are attached in the supplementary information^46^.

For dynamic analysis, the deuterium differences were then mapped onto representative Beta structures (PDB: 7LYQ). For epitope mapping of mAb 511, the deuterium differences were mapped on representative ancestral structure (PDB: 6VSB).

### Supplementary Information

Supplementary Information.

Supplementary Figures.

## Data Availability

Atomic models described in this study and single particle cryo-EM maps have been deposited to the Protein Data Bank (PDB)/Electron Microscopy Data Bank (EMDB), under the following accession codes: Ancestral antigen (PDB: 8TYL/EMDB-41725) and Beta antigen (PDB: 8TYO/EMDB-41727). Previously published structural models cited in the paper include the PDB entries 6VYB and 7LYK. Summary of all the peptides for Beta spike antigen used for the hydrogen exchange experiments and analysis parameters are included as supplementary csv files. Raw mass spectrometry data is available upon request (Eduardo Bruch).
