# Supplementary material for: Structural and biochemical rationale for Beta variant protein booster vaccine broad cross-neutralization of SARS-CoV-2
Source: Sci Rep. 2024 Jan 23;14:2038. doi: 10.1038/s41598-024-52499-1 (PMC10805794; doi:10.1038/s41598-024-52499-1)
Supplement: Supplementary file 1 — Supplementary Information. [file 41598_2024_52499_MOESM1_ESM.docx]

**``````````````````````````Supplementary information**

**Supplementary Note**

Ancestral trimer structural description and S1 comparison between ancestral and Beta trimers

Model building for the ancestral trimer was performed using 6vyb as the starting model^16^. The map shows clear density for the S2 region from residues 690 to 1140. Consistent with the reported structure from Wrapp et al^16^, only two loops lack defined structural density. These loops correspond to the S1/S2 transition (residues 677 to 689) and the FPPR region (residues 828 to 853). A clear difference between the model and 6vyb is the absence of the C-terminal trimerization helix where no density is observed in our map (Figure 2A and B). Another difference is the orientation of the K854 side chain that in our model faces D614, providing evidence for the role of the D614-K854 salt bridge, although the density is only clear within the chain A-chain B salt-bridge (Figure 2F). The Beta trimer model was built starting from the two_RBD up conformation structure 7lyk^21^. The model is clearly defined in the S2 region, it includes residues from 689 to 1140 with only two missing regions corresponding to a loop in the S1/S2 transition (residues 677-688) and within the FPPR region (residues 828-854). Unlike the reference structure, the C-terminal helix (residues 1141-1146) could not be built because no density was observed.

In the S1 region, the ancestral trimer model includes residues 27 to 676. The NTD, mostly formed of β-sheets, includes residues 27 to 305 with gaps corresponding to disordered loops. The NTD-RBD linker region is clearly visible (residues 305 to 330). Both RBD in the down conformation could be modelled with the exception of two sequences in the more distal region (443-451 and 471-502 for Chain A and 445-460 and 467-490 for Chain C). For chain B where the RBD is in the up conformation, the RBD could not be modelled, contrasting the results reported in Wrapp et al ^16^(Figure 2B). The 630 loop was clearly visible in chain B and C, but almost no density was observed for this loop in chain A. This is in agreement with the position of the nearest RBD, with more define density for the 630 loop if the neighbouring RBD is in a down conformation. (Figure 2D and E). The S1 sequence coverage of the Beta trimer model changes between the protomers since for both up oriented RBD chains the corresponding RBD was excluded from the model due to poor density (Figure 3B and Supplementary Figure 5C). All NTDs show clear density for the β-sheets with four loops that are not defined. The SD1/SD2 region is clearly visible with only a minor gap corresponding to the 630 loop, as previously described^44^.The mutations in the NTD were found in linker regions that were not visible in the Beta trimer model. An exception is D215G, this mutation is located within a linker region that is visible for all three protomers. Moreover, for the protomer where the NTD is next to the down oriented RBD the latter stabilizes this linker region resulting in a more open linker with clearer density (Figure 2C and 3C). The D614G mutation is located within the SD1/SD2 interface and eliminates a salt bridge between D614 and K854 in the FPPR of the adjacent protomer, the position of K854 in our model differs from the one reported previously. Within the S2 both the A701V and the 2xPro mutations could be modelled with clear densities (Figure 3 and Supplementary Figure 5C) for the Beta trimer. Finally, Asn-linked N-acetylglucosamines were modelled for Asn residues 282, 331, 616, 657, 709, 717, 801, 1074, 1134 and 1098.

The binding epitope of antibody 511 was determined by HDX-MS using the ancestral strain and the resulting epitope was determined to be residues 969-988 (containing) the 2x proline mutation as this region is solvent exposed from a structural perspective (Supplementary Figure 6). Binding sites for ACE2 and epitopes of CR3022 and 511 are summarized in Table 1.

Supplementary Figures


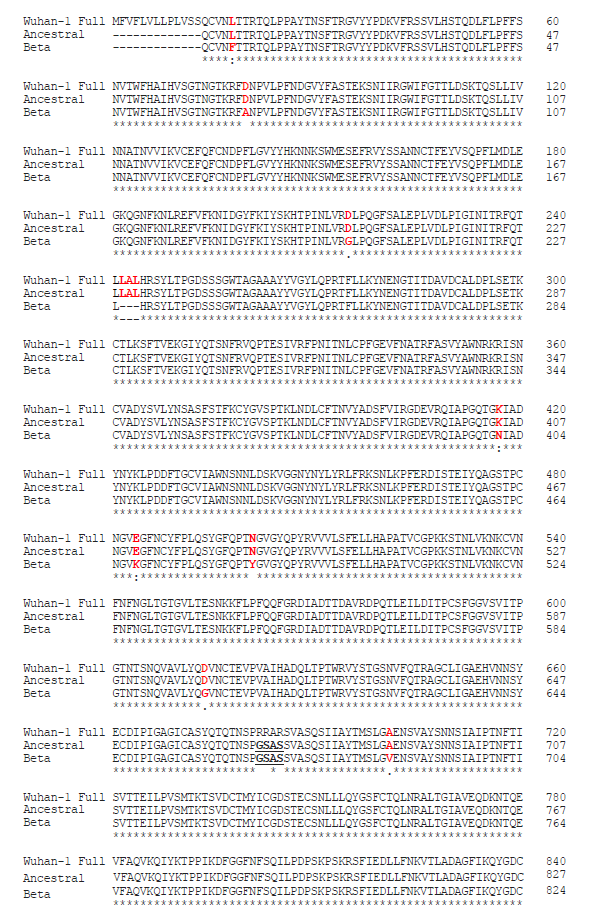


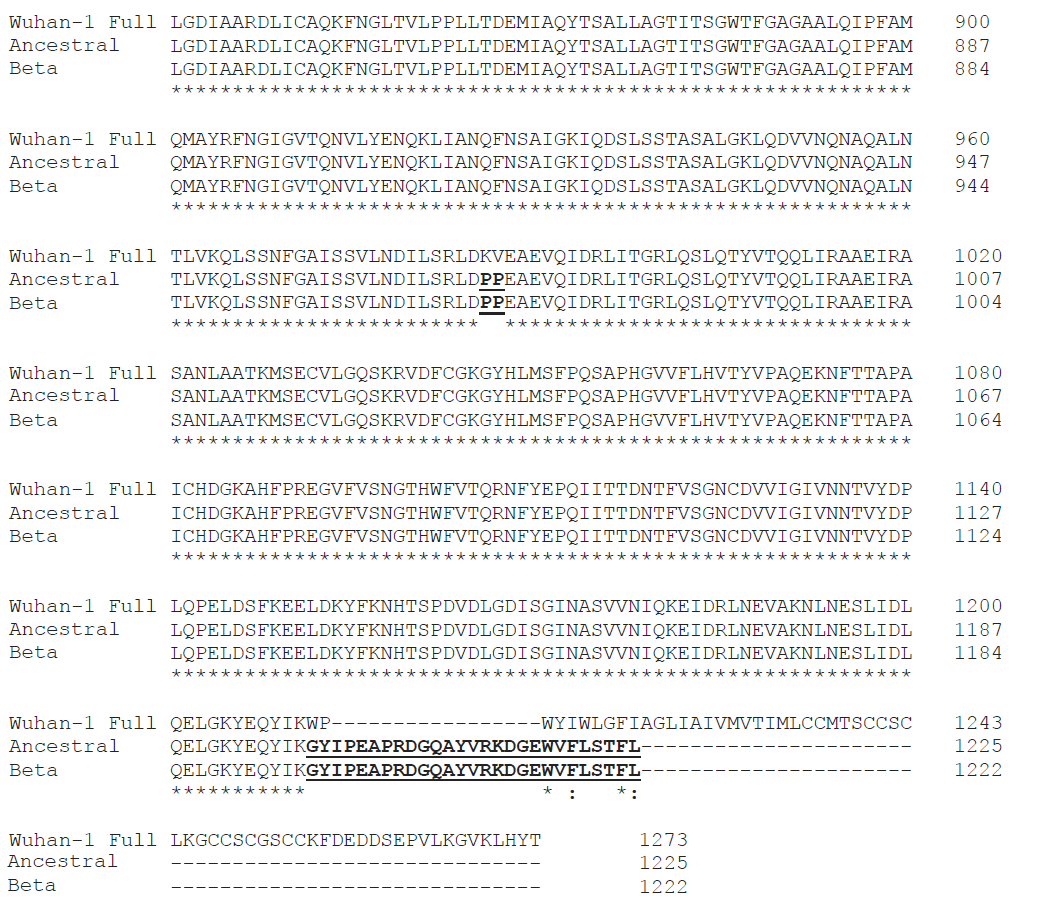


**Supplementary Figure 1. Amino acid comparison between full-length Wuhan Hu-1 SARS-CoV-2 spike antigen with mature coding for both the recombinant ancestral and Beta spike antigens.** The amino acid sequence of the signal sequence, modification to the furin protease cleavage site, stabilizing prolines, and the C-terminal T4 bacteriophage fibritin foldon domain are underlined. Point mutations and deletions between the ancestral and Beta spike antigens are shown in red. The amino acid sequence for the mature protein is presented for recombinant ancestral and Beta spike antigens and the numbering of the deletions and point mutations is based on the Wuhan Hu-1 full-length protein sequence.


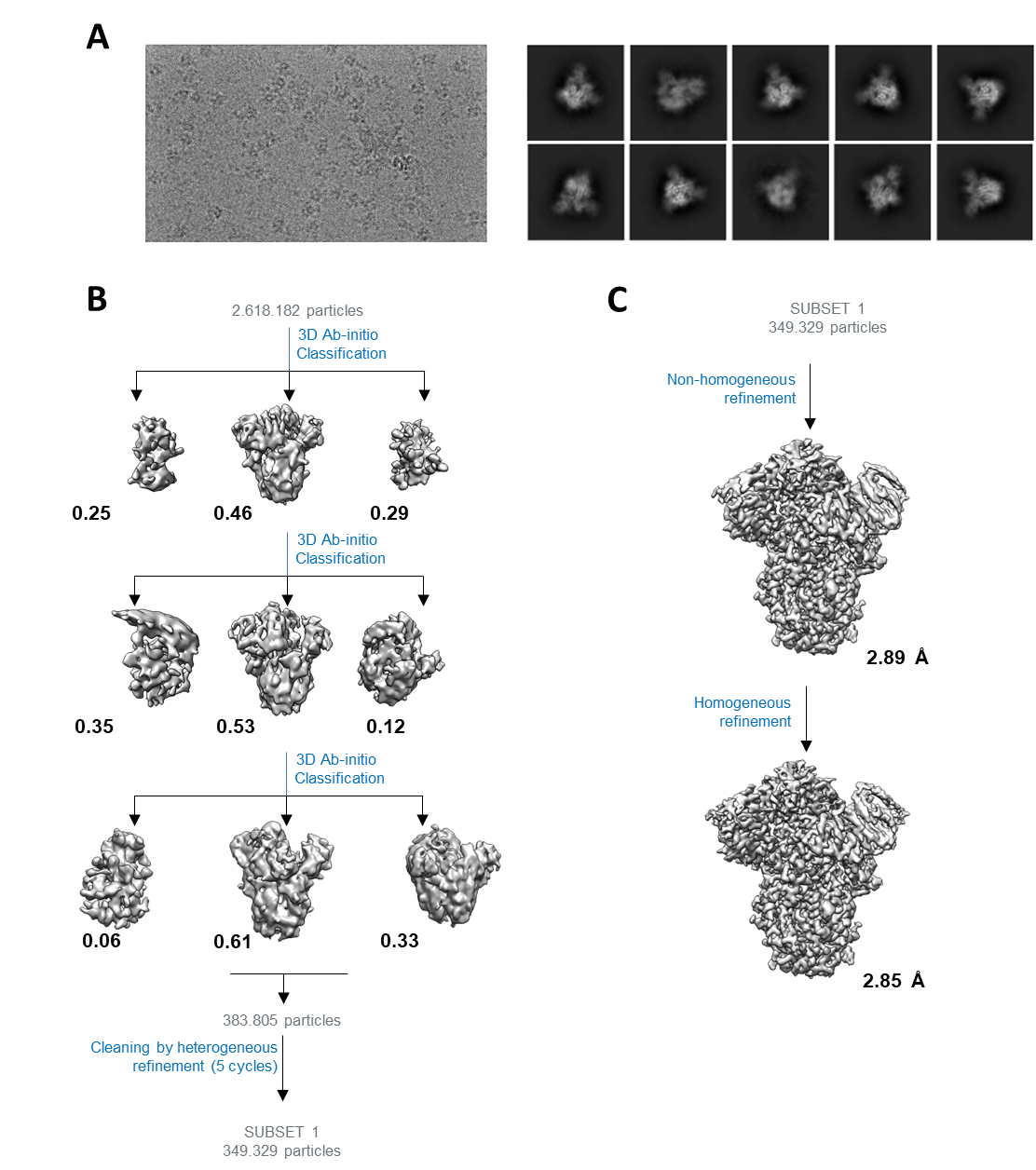


**Supplementary Figure 2. Ancestral trimer cryo-EM data analysis pipeline. (A)** Representative micrograph and 2D-classes. **(B)** Particle cleaning using several rounds of Ab-initio 3D classification followed by heterogeneous refinement cycles. **(C)** The final particle dataset was refined using non-uniform refinement followed by homogeneous refinement reaching a final resolution of 2.89 and 2.85 respectively.


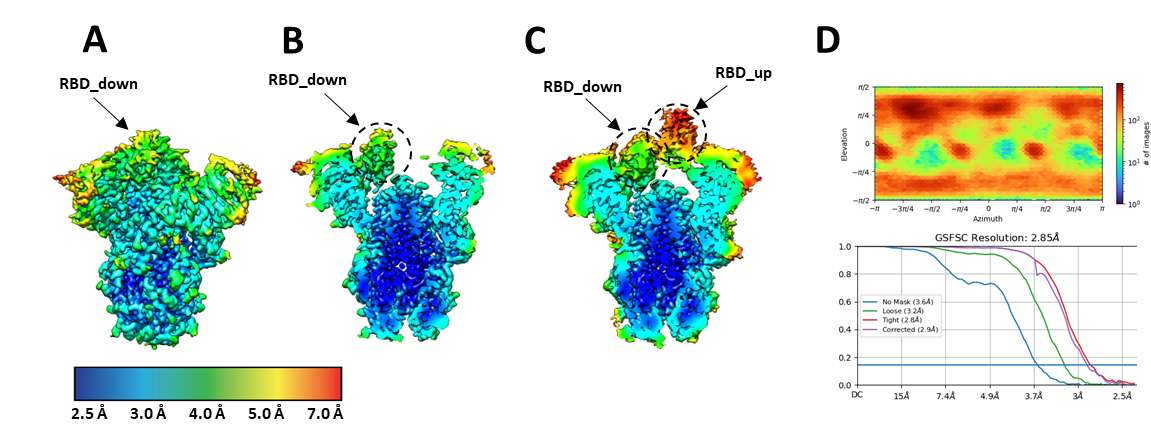


**Supplementary Figure 3: Analysis of overall and local map resolution (A)** Cryo-EM map of the ancestral trimer coloured according to local resolution estimation by locres. The electron density threshold was set to a value where the density is good for model building (the RBD up density is not present at this threshold). **(B)** Sliced volume showing the different local resolution when comparing S2 core and S1 distal domains. **(C)** Same region with decrease electron density threshold. Regions with weaker density and lower associated resolution such as the RBD up domain became visible. **(D)** Particle distribution and overall map resolution for the final map refinement.


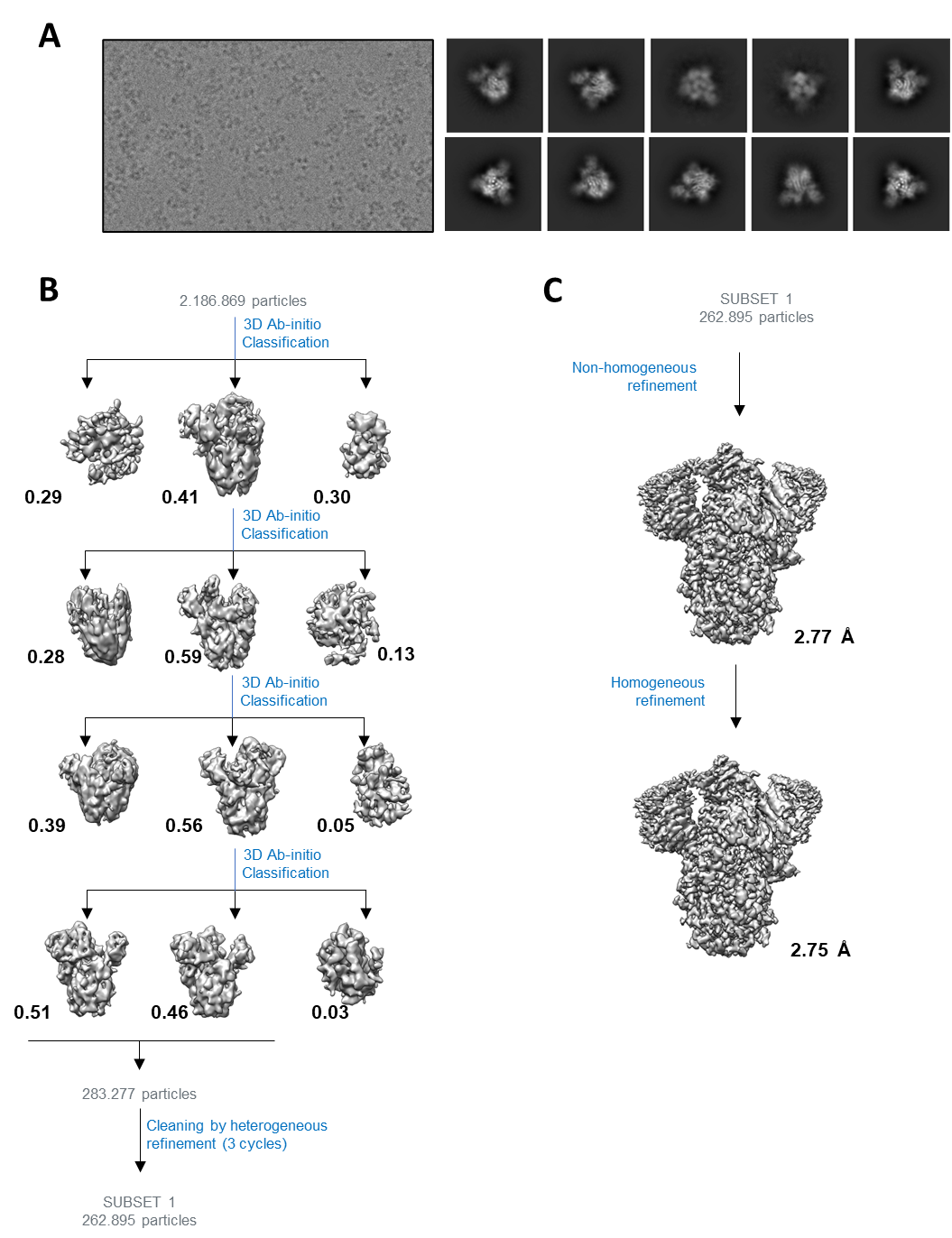


**Supplementary Figure 4. Beta trimer cryo-EM data analysis pipeline. (A)** Representative micrograph and 2D-classes. **(B)** Particle cleaning using several rounds of Ab-initio 3D classification followed by heterogeneous refinement cycles. **(C)** The final particle dataset was refined using non-uniform refinement followed by homogeneous refinement reaching a final resolution of 2.89 Å and 2.85 Å, respectively.


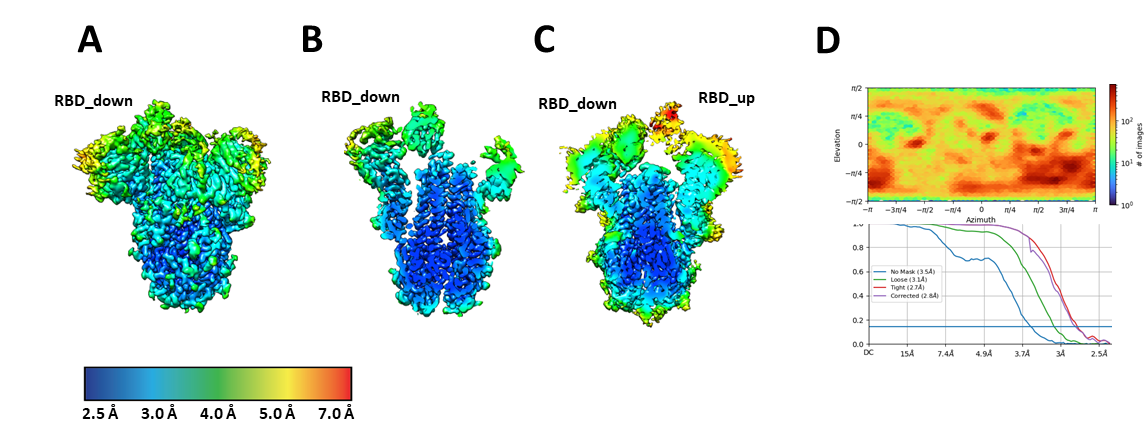


**Supplementary Figure 5: Analysis of overall and local map resolution for Beta trimer. (A)** Cryo-EM map of the Beta trimer coloured according to local resolution estimation by locres. The electron density threshold was set to a value where the density is good for model building (the RBD up density is not present at this threshold). **(B)** Sliced volume showing the different local resolution when comparing S2 core and S1 distal domains. **(C)** Same region with decrease electron density threshold. Regions with weaker density and lower associated resolution such as the RBD up domain became visible. **(D)** Particle distribution and overall map resolution for the final map refinement.


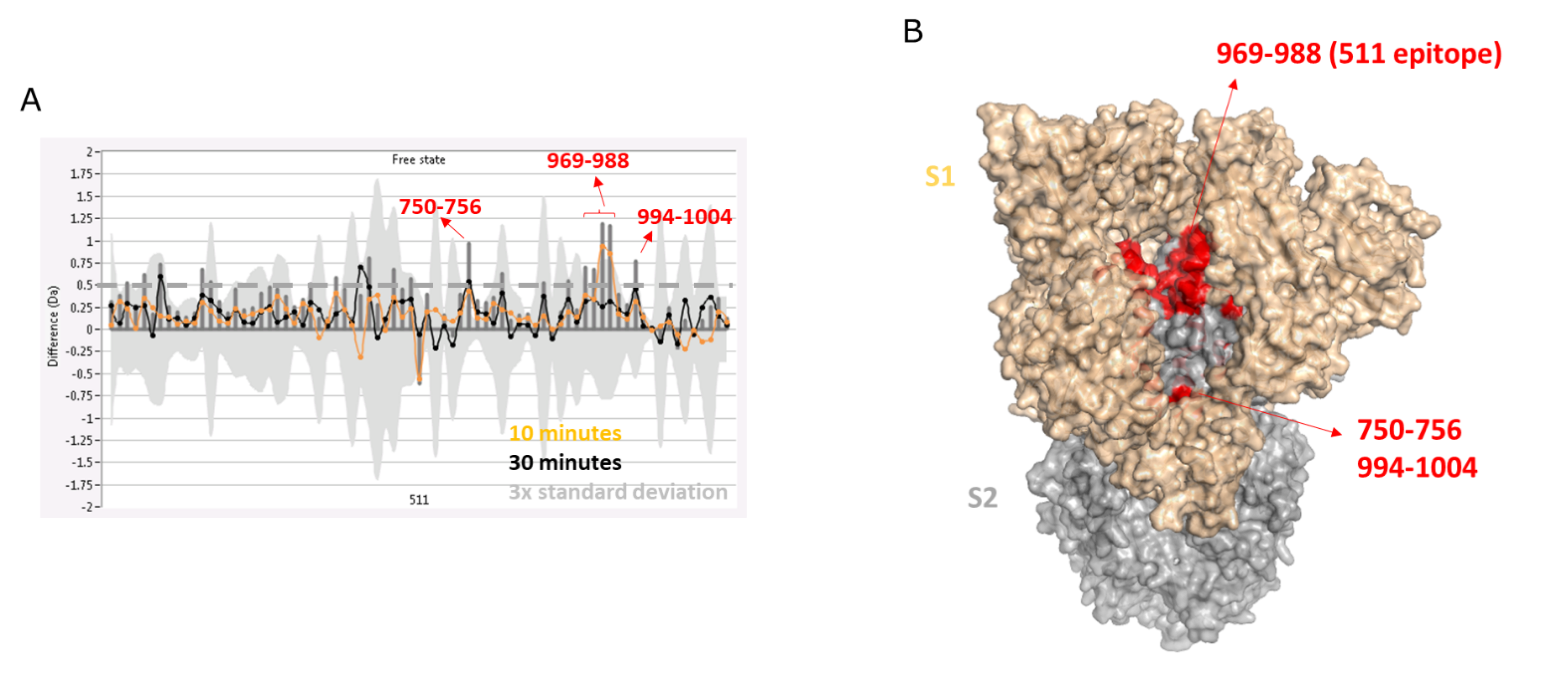


**Supplementary Figure 6. Epitope characterization of antibody 511. (A)** Difference plot between ancestral spike antigen and ancestral spike/antibody 511 complex. Y-axis is the deuterium difference and X-axis is peptides ordered sequentially from N- to C-terminus. Orange and black trace is the deuterium difference data for 10 and 30 minutes of HDX-MS mixing time, respectively. Individual bars represent summed difference. Grey shaded region is 3 times standard deviation, and the grey dotted line is the 0.5 Da threshold. Peptides with significant decrease in deuterium difference is highlighted in red. **(B)** HDX-MS difference data mapped onto representative structure (PDB: 6VSB). S1 and S2 domains are highlighted in wheat and grey colours, respectively. The three regions, 750-756, 969-988, and 994-1004, that exhibited significant difference is shown in red. Epitope of antibody 511 is residues 969-988.
