# Supplementary material for: Structural and biochemical rationale for Beta variant protein booster vaccine broad cross-neutralization of SARS-CoV-2
Source: Sci Rep. 2024 Jan 23;14:2038. doi: 10.1038/s41598-024-52499-1 (PMC10805794; doi:10.1038/s41598-024-52499-1)
Supplement: Supplementary file 2 — Supplementary Figures. [file 41598_2024_52499_MOESM2_ESM.docx]

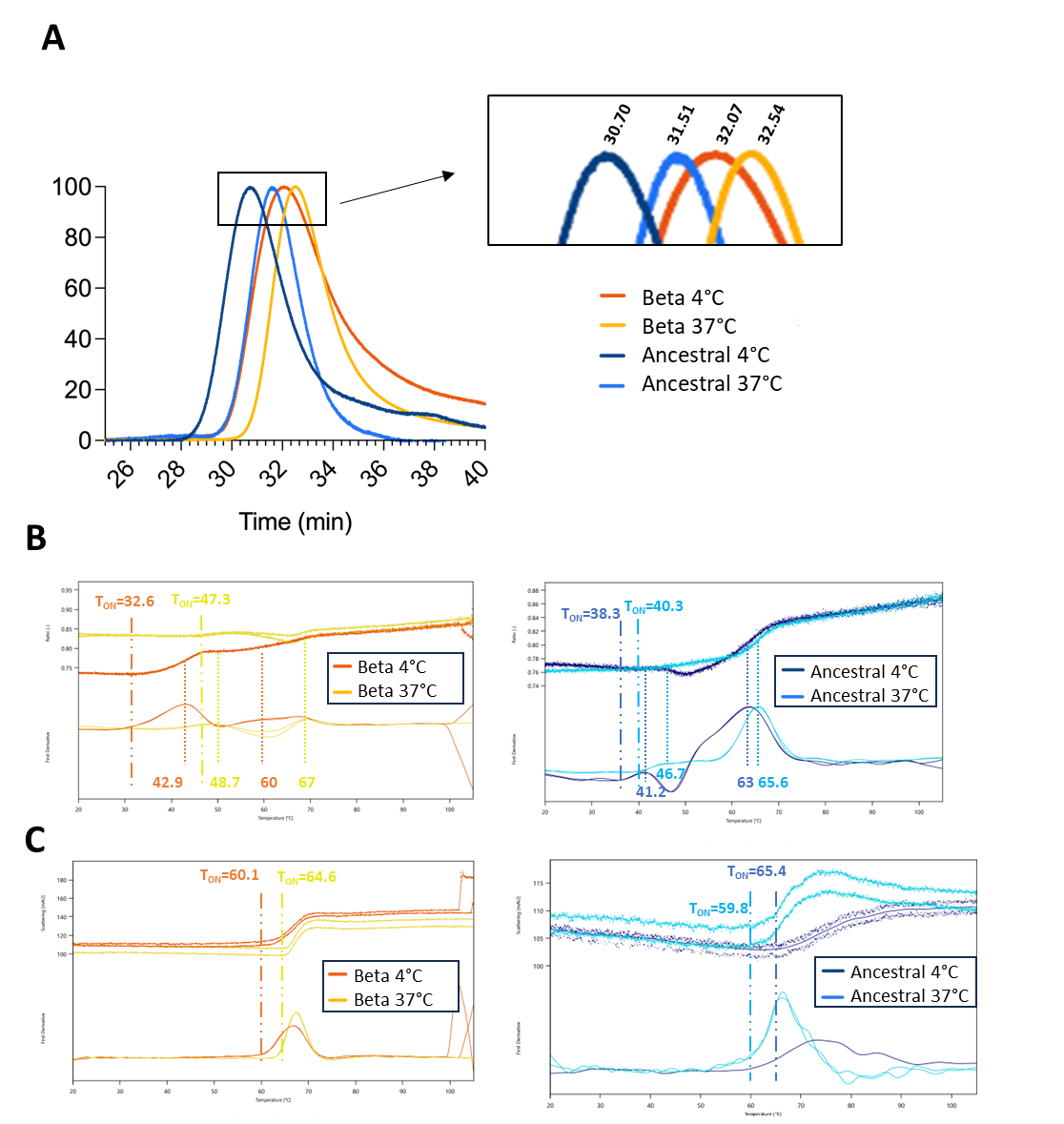


**Extended Data Figure 1** **Biophysical characterization of cold stored vs temperature treated antigens. (A)** Size exclusion chromatography elution of cold stored and temperature treated ancestral and Beta spike antigens (orange, yellow, blue and cyan respectively). The elution times for all samples are shown in the INSET. **(B)** nanoDSF analysis showing the 350/330 ratio for the thermal unfolding curves (top) and its first derivative (bottom) for Beta spike antigen (left) and ancestral spike antigen (right) by duplicate. **(C)** nanoDSF analysis showing the scattering curves for the thermal unfolding (top) and its first derivative (bottom) for Beta spike antigen (left) and ancestral spike antigen (right) by duplicate.


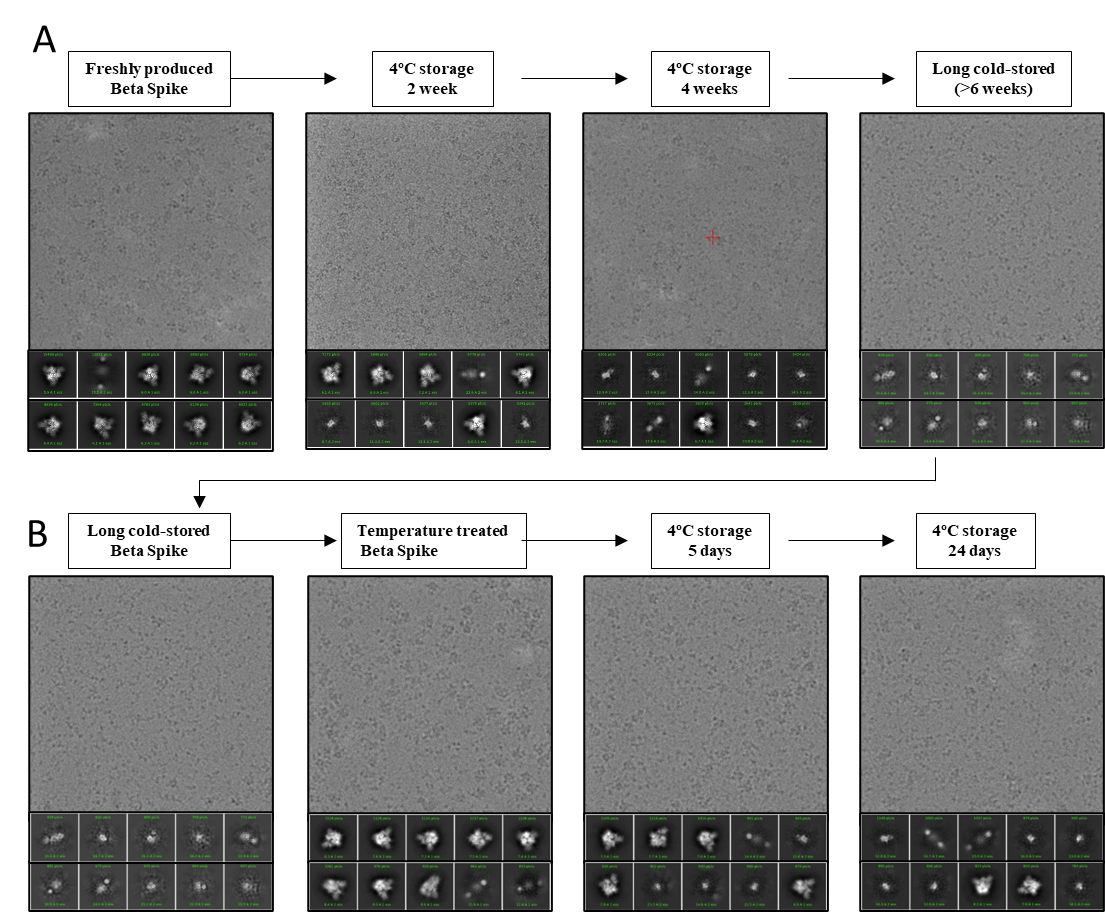


**Extended Data Figure 2. Effect of cold storage and temperature treatment in Beta spike antigen. (A)** Cryo-EM representative micrograph and the ten most populated 2D-classes are shown for a freshly produced Beta trimer (left) and after cold storage for two, four and more than six weeks (ordered from left to right). **(B)** Cryo-EM representative micrograph and the ten most populated 2D-classes are shown for: a sample cold stored for several weeks, after temperature treatment and after cold storage for five and 24 days (ordered from left to right).
